# Redox changes of ferredoxin, P700, and plastocyanin measured simultaneously in intact leaves

**DOI:** 10.1007/s11120-017-0394-7

**Published:** 2017-05-11

**Authors:** Ulrich Schreiber

**Affiliations:** Julius-von-Sachs Institut für Biowissenschaften, Universität Würzburg, Julius-von-Sachs Platz 2, 97082 Würzburg, Germany

**Keywords:** Chlorophyll fluorescence, C3 and C4 photosynthesis, Cyclic electron transport, Difference spectroscopy, Dual/KLAS-NIR spectrophotometer, Light activation

## Abstract

Properties and performance of the recently introduced Dual/KLAS-NIR spectrophotometer for simultaneous measurements of ferredoxin (Fd), P700, and plastocyanin (PC) redox changes, together with whole leaf chlorophyll a (Chl) fluorescence (emission >760, 540 nm excitation) are outlined. Spectral information on in vivo Fd, P700, and PC in the near-infrared region (NIR, 780–1000 nm) is presented, on which the new approach is based. Examples of application focus on dark–light and light–dark transitions, where maximal redox changes of Fd occur. After dark-adaptation, Fd reduction induced by moderate light parallels the Kautsky effect of Chl fluorescence induction. Both signals are affected analogously by removal of O_2_. A rapid type of Fd reoxidation, observed after a short pulse of light before light activation of linear electron transport (LET), is more pronounced in C4 compared to C3 leaves and interpreted to reflect cyclic PS I (CET). Light activation of LET, as assessed via the rate of Fd reoxidation after short light pulses, occurs at very low intensities and is slowly reversed (half-time ca. 20 min). Illumination with strong far-red light (FR, 740 nm) reveals two fractions of PS I, PS I (LET), and PS I (CET), differing in the rates of Fd reoxidation upon FR-off and the apparent equilibrium constants between P700 and PC. Parallel information on oxidation of Fd and reduction of P700 plus PC proves essential for identification of CET. Comparison of maize (C4) with sunflower and ivy (C3) responses leads to the conclusion that segregation of two types of PS I may not only exist in C4 (mesophyll and bundle sheath cells), but also in C3 photosynthesis (grana margins plus end membranes and stroma lamellae).

## Introduction

Ferredoxin (Fd) is located at the ‘end of the line’ of the photosynthetic light reactions (Goss and Hanke 2014), playing a pivotal role as distributor of electrons originating from the splitting of water in photosystem II (PS II) between several metabolic pathways at the acceptor side of photosystem I (PS I). Within the PS I core complex charge separation is stabilized via a cascade of rapid electron transfer reactions in the ps to ns time range from P700 via A_0_ (monomeric form of Chl a) and A_1_ (phylloquinone) to the [4Fe–4S] centers F_x_, F_A_, and F_B_, with the latter corresponding to P430 of Hiyama and Ke (1971). F_B_ is the distal stromal FeS cluster that transfers electrons to Fd, a small soluble [2Fe–2S] cluster (Vassiliev et al. 1998; Diaz-Quintana et al. 1998). In the absence of oxidized Fd or any exogenous electron acceptor, the formation of the charge separated state P700^+^ F_B_^−^ is followed by charge recombination occurring in the 10–100 ms time range (Brettel 1997).

Fd can bind with high affinity to the stromal side of PS I (Sétif 2001), serving as one-electron carrier (E_m_ ~ −420 mV). Besides binding close to F_B_ to the PS I complex, soluble Fd can also bind to various stromal enzymes, thus channeling the electrons received via PS I charge separation into various metabolic pathways. These include the ferredoxin–NADP reductase (FNR) catalyzed reduction of NADP (Shin et al. 1963; Carillo and Ceccarelli 2003), reduction of O_2_ and H_2_O_2_ in the Mehler-ascorbate-peroxidase (MAP) cycle (Asada and Badger 1984; Schreiber et al. 1995; Asada 1999; Miyake 2010), nitrite reduction (Anderson and Done 1978), various types of cyclic electron transport (CET) (Arnon and Chain 1975; Bendall and Manasse 1995; Miyake et al. 1995; Munekage et al. 2002; Joliot and Joliot 2006; Shikanai 2007; Laisk et al. 2010), and reduction of thioredoxin, the key redox regulator of numerous processes at various levels of chloroplast metabolism (Buchanan 1980; Knaff 1996; Buchanan et al. 2002).

In spite of its key role in photosynthesis, until very recently no direct measurements of Fd redox changes in vivo have been reported. As pointed out by Bacon Ke, one of the most prominent researchers on PS I, the “absorption spectra of both oxidized and reduced Fd in the blue region are rather featureless and their extinction coefficients are also relatively low” and, therefore, “direct spectrophotometric measurement of electron transfer from PS I to Fd has generally not been attempted by measuring changes in its own absorbance” (Ke 2001). Furthermore, the visible absorption bands of Fd overlap with those of chlorophylls, P700, P430, etc., whose absorbance changes can readily overwhelm that by Fd itself. Some of these difficulties could be overcome by in vitro measurements of 480 nm absorbance using PS I particles to which exogenous Fd was added (Hervas et al. 1992; Sétif and Bottin 1995). While such measurements have resulted in important insights on the binding of Fd to the stromal side of PS I (Sétif 2001), they could not readily be extended on the in vivo system for studying the complex reactions downstream of Fd.

Since the introduction of PAM fluorometry (Schreiber 1986) and of devices capable of monitoring P700-related absorbance changes in intact leaves (Weis et al. 1987; Harbinson and Woodward 1987; Schreiber et al. 1988), an extensive body of experimental data on photosynthetic reactions in vivo has accumulated, a large part of which *indirectly* reflects the reactions of Fd. This is particularly true for measurements of dark–light induction kinetics, where Fd acts like a bottle neck in the flow of electrons from PS II via PS I to NADP and various alternative acceptors. In this way, *indirect* evidence for various pathways of Fd-mediated cyclic electron transport around PS I (CET) has been obtained (Mi et al. 1992; Asada et al. 1993; Joliot and Joliot 2002, 2005, 2006; Munekage et al. 2002; Golding and Johnson 2003; Joliot et al. 2004; Chow and Hope 2004; Laisk et al. 2005, 2010; Breyton et al. 2006; Talts et al. 2007; Fan et al. 2007; Joliot and Johnson 2011).

When in the past conclusions on Fd-mediated reactions were drawn from measurements of P700, there was always some uncertainty about potential interference of plastocyanin (PC), which is known to show absorbance changes in the same 810–830 nm spectral region where normally P700 is measured (Klughammer and Schreiber 1991). For example, in their study on the regulation of cyclic and linear electron flow in higher plants, Joliot and Johnson (2011) measured P700 via absorbance changes at 820 nm, mentioning that “at this wavelength, P700 and PC oxidation contribute 77 and 23% to the signal, respectively,” assuming that absorption changes at 820 nm can be “considered as giving an acceptable approximation of P700 redox state.” While this may hold for some conditions, it certainly cannot be assumed to be generally true. As will be shown in the present communication, there are situations where almost fully reduced P700 coexists with PC being 30–50% oxidized.

Here *direct* measurements of deconvoluted redox changes of Fd, P700, and PC will be reported on, using a prototype of a newly developed device, the Dual/KLAS-NIR spectrophotometer. Technical details of the new device were already outlined in a preceding ‘emerging techniques’ article (Klughammer and Schreiber 2016) and some examples of applications were previously presented (Schreiber and Klughammer 2016). First some more background information will be given on the spectral properties of Fd, P700, and PC in the NIR, on which deconvolution is based. Then experimental data on dark–light induction and light–dark relaxation kinetics will be presented, where Fd redox changes are most pronounced and can be quantitatively analyzed. Particular attention will be given to the process of light activation and to rapid antiparallel changes of Fd oxidation and PC reduction after pulse illumination which are interpreted to reflect a rapid pathway of cyclic electron transport (CET). It will be shown that part of Fd capable of rapid CET can coexist with another part of Fd that cannot readily be reoxidized by the PS I donor side. Comparison of the responses obtained with C3 and C4 leaves will lead to the conclusion that two types of PS I, specialized in linear electron transport (LET) and CET, not only occur in the chloroplasts of mesophyll cells (MC) and bundle sheath cells (BSC) of C4 leaves, but also in segregated domains of C3 chloroplasts, as originally suggested by Anderson (1982) and Albertsson (1995).

## Materials and methods

### Dual/KLAS-NIR spectrophotometer

The Dual/KLAS-NIR spectrophotometer is the most recent member of a family of pulse-amplitude-modulated (PAM) devices, development of which was initiated more than 30 years ago, first for measuring Chl fluorescence (Schreiber 1986; Schreiber et al. 1986), then 830 nm absorbance changes for assessment of P700 (Schreiber et al. 1988), and then also absorbance changes in the green spectral region for differentiation of cytochrome redox changes from the much larger overlapping changes caused by P515, ‘light scattering,’ and zeaxanthin (Klughammer et al. 1990). While the Dual-PAM-100 for simultaneous measurements of Chl fluorescence and P700 has been widely used by photosynthesis researchers, the KLAS-100 (kinetic LED array spectrophotometer) until to date has remained a device for specialists.

The new Dual/KLAS-NIR combines basic features of Dual-PAM-100 and KLAS-100. The Dual-PAM-100 measures the difference between two transmission pulse signals at 820 and 870 nm, thus largely eliminating the contribution of PC, so that deconvolution can be avoided. This means, however, that no information on PC is obtained. The KLAS-100 measures 8 difference signals with the aim of deconvoluting the changes of cyt f, cyt b_6_, cyt b559, C550, P515 (electrochromic absorbance shift), ‘light scattering,’ and zeaxanthin. The new Dual/KLAS-NIR measures four difference signals in the near-infrared (NIR) spectral region, which carry sufficient information for deconvolution of Fd, P700, and PC (Klughammer and Schreiber 2016). In addition, it can simultaneously measure two types of Chl fluorescence, with 540 and 440 nm excitation. In the present study, use of 540 nm excitation was made, with which fluorescence throughout the whole leaf is measured. The same photodiode detector protected by 1 mm RG9 plus 1 mm RG780 filters (Schott) was applied for both fluorescence and NIR transmittance measurements. In this way, light-induced changes of Fd, P700, and PC could be directly compared with fluorescence changes measured under identical optical conditions.

### Redox difference spectra in the NIR of Fd, P700, and PC in intact leaves

**Fig. 1:**
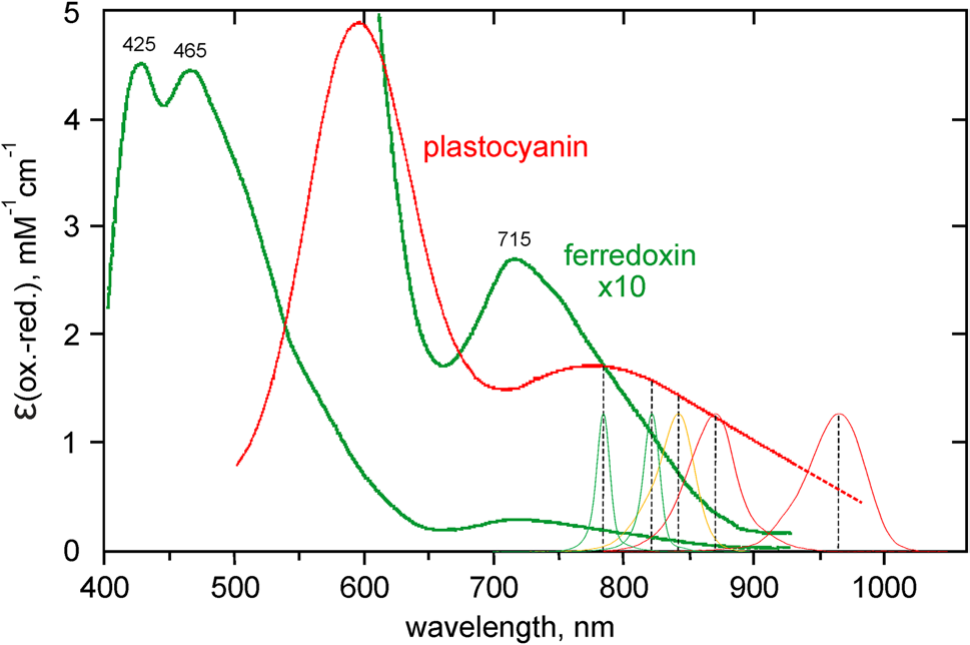
Oxidized minus reduced extinction coefficients of isolated PC and Fd. Reproduced from Klughammer (1992). In the NIR spectral range, the spectra of the 5 types of measuring light are inserted (785, 820, 840, 870, and 965 nm). The half-band widths of 785, 820, and 840 nm measuring light are narrowed down to 8, 10, and 23 nm with the help of miniature interference filters. The Dual/KLAS-NIR prototype applied in the present study measures the difference signals 785–820, 820–870, 870–965, and 840–965 nm

Measuring redox difference spectra of Fd, P700, and PC in intact leaves is not an easy task considering that the light-induced redox changes of these closely linked electron carriers normally overlap. Such measurements are feasible at wavelengths above 780 nm only, where the contributions of Chl fluorescence and of an electrochromic absorbance shift peaking at 740 nm are negligibly small (Klughammer 1992). In Fig. 1, the oxidized minus reduced absorbance difference spectra of *isolated* Fd and PC in vitro are shown. The major absorption peaks of both components are located in the visible region. At wavelengths >780 nm, the extinctions of both components decline, with extinction of Fd being about 10× lower than that of PC and showing a distinctly faster decline.

During the development of the Dual/KLAS-NIR, the optimal choice of the wavelength pairs for the four difference signals has been a process of trial and error. As initially no information on redox difference spectra in intact leaves was available, the wavelengths were chosen on the basis of the in vitro spectra shown in Fig. 1 and for P700 from far-red-induced changes in intact spinach leaves (Schreiber et al. 1988) as well as light-induced changes in chemically treated isolated chloroplasts (Klughammer 1992, see also Supplementary Fig. 2 of Klughammer and Schreiber 2016). Measurements presented in the present communication were carried out with a prototype featuring the wavelength pairs 785–820, 820–870, 870–965, and 840–965 nm. The peak positions and bandwidths of the chosen measuring wavelengths are indicated in Fig. 1.

**Fig. 2:**
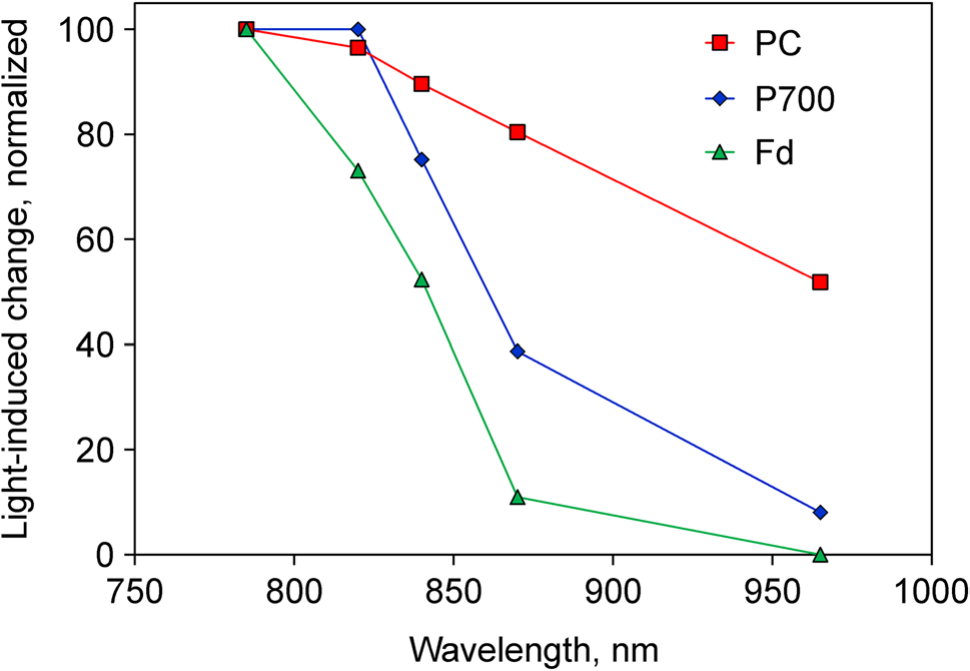
Redox difference spectra of Fd (*green*), P700 (*blue*), and PC (*red*) measured with intact sunflower leaf. Single wavelength transmittance changes were induced under conditions favoring selective redox changes of either Fd or P700 or PC (see legend to Supplementary Fig. 4 in Klughammer and Schreiber 2016). Maximal oxidized minus reduced (Fd) and reduced minus oxidized (P700 and PC) transmittance changes were normalized to 100 units

In vivo redox difference spectra of an intact sunflower leaf measured with the Dual/KLAS-NIR are presented in Fig. 2. While this instrument normally is operated in the dual-wavelength difference mode, these spectra were recorded in the single wavelength mode by lowering the LED pulse currents of the respective dual-wavelength partners to zero. With difference signals saturating around 4 V, for measurement of these spectra the single signal amplitudes, which normally amount to 100–200 V, had to be substantially decreased by lowering measuring light intensity and amplifier gain. Selective redox changes of Fd, P700, and PC were induced as described in Klughammer and Schreiber (2016) and Schreiber and Klughammer (2016).

The following conclusions can be drawn from the in vivo difference spectra in Fig. 2:

1. While all three components display declining extinction in the 785–965 nm range, there are distinct differences in the steepness of the three declines, with Fd>P700>PC.
2. The 785–820 and 820–870 nm difference signals are dominated by Fd and P700, respectively, whereas 870–965 nm contains relatively much PC. The 840–965 nm difference signal contains large amplitudes of all three components which is advantageous with regard to a high signal/noise ratio.

In normal practice, using the Dual/KLAS-NIR not redox difference spectra of single wavelength signals, but rather redox difference spectra of dual-wavelength difference signals are measured at considerably enhanced sensitivity (see above). The obtained spectral information, which is plotted in the so-called ‘model spectra’ or ‘differential model plots’ of Fd, P700, and PC, is the basis for the computer-assisted deconvolution of mixed time-dependent redox changes of Fd, P700, and PC (Klughammer and Schreiber 2016).

### Software-controlled routine steps for measuring simultaneous redox changes of Fd, P700, and PC

In the present study, for all leaf samples the same standard ‘model spectra’ for Fd, P700, and PC were used. For every new leaf sample, the following software-controlled routine steps were carried out:

- balancing the two signals of each of the four wavelength pairs, so that all four difference signals were zeroed;
- calibration of the signal amplitudes, after which changes in difference signals were displayed in units of ΔI/I (changes of transmittance);
- deconvolution of the difference signals, after which the contributions of Fd, P700, and PC to the overall transmittance changes were displayed in units of ΔI/I (fractional transmittance changes corresponding to relative redox changes);
- induction of maximal redox changes of Fd, P700, and PC by a special illumination program (script-controlled so-called NIRmax measurement); and
- determination of the 100% changes of Fd, P700, and PC (Get NIRmax routine), after which the deconvoluted signals of Fd, P700 and PC could be displayed in absolute units of % oxidation (Klughammer and Schreiber 2016).

### Plant material

Sunflower (*Helianthus annuus*) was grown in a sun-exposed private garden. Maize (*Zea mays*) was obtained from the south edge of a maize field in the vicinity of Sommerhausen (Frankonia). Ivy leaves (*Hedera helix*) were collected from a north-west-exposed house balcony. Leaves of *Amaranthus retroflexus* were collected from a vineyard close to Würzburg–Heidingsfeld.

### Cuvette and sample preparation

After cutting the petioles of harvested leaves under water, the detached leaves were kept fully turgescent at a north window in natural daylight for several days. For determination of 100% redox changes, leaves were dark-adapted for at least 3 h. Leaf samples were enclosed in a Dual-PAM gas-exchange Cuvette (3010-Dual, Heinz Walz GmbH) with temperature control and flushed with a stream of either humidified air or humidified, CO_2_-saturated N_2_. All experiments were carried out at 25 °C.

## Results and interpretation

### Dark–light-induced redox changes of PS I electron carriers and Chl fluorescence

For comparison of the deconvoluted redox changes of Fd, P700, and PC with Chl fluorescence changes, it was important that fluorescence originated from the same chloroplasts in which the monitored Fd, P700, and PC was located. Therefore, 540 nm pulse-modulated measuring light (FluoML) was applied, which excites all leaf layers evenly, and fluorescence was measured at wavelengths >760 nm, i.e., without significant reabsorption, so that measured fluorescence was representative for the whole leaf.

**Fig. 3:**
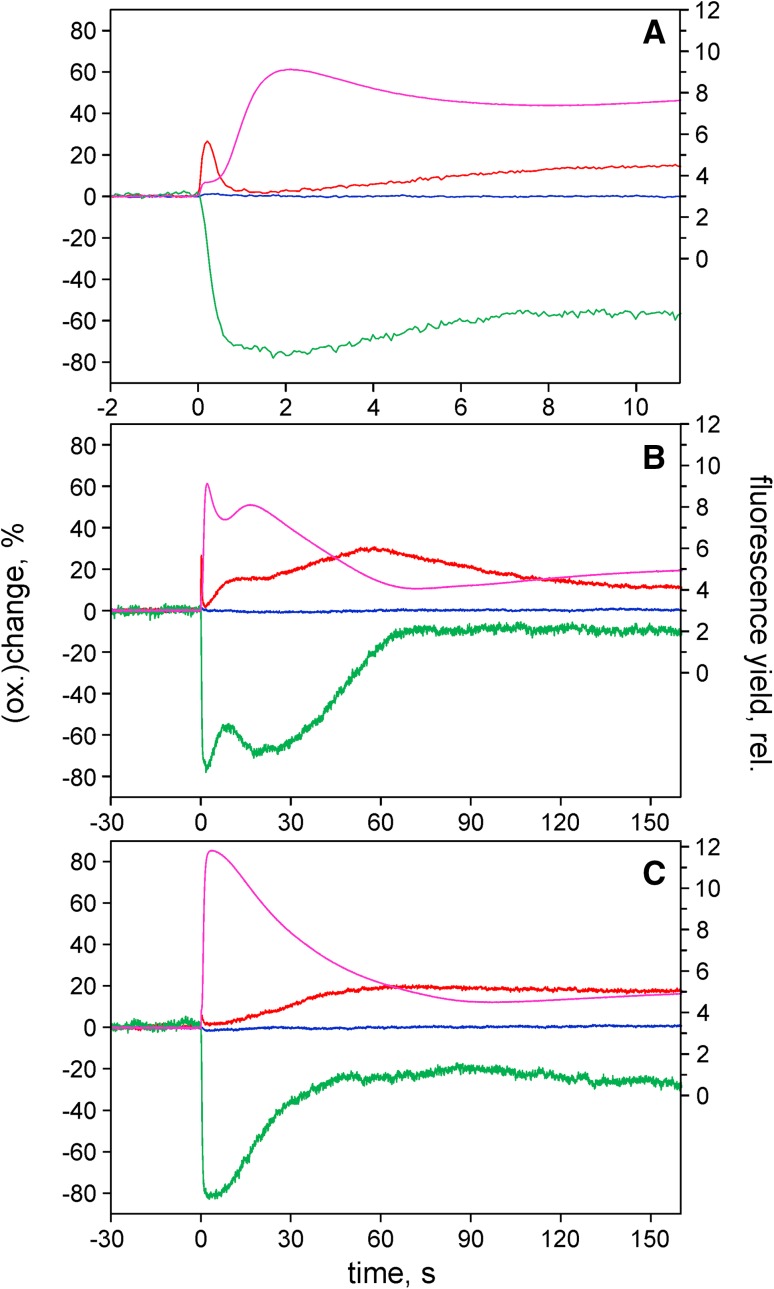
Redox changes of Fd (*green*), P700 (*blue*), and PC (*red*) measured simultaneously with Chl fluorescence (*magenta*) upon a dark–light transition with an intact sunflower leaf. Intensity of 630 nm actinic light, 60 µmol m^−2^ s^−1^. **a** Rapid transients, air. **b** Slow transients, air. **c** Slow transients, N_2_ + CO_2_. Whole leaf fluorescence >760 nm excited with 540 nm pulse-modulated measuring light. Single measurements

Figure 3 shows typical dark–light induction transients of Fd (green), PC (red), and Chl fluorescence (magenta) in air (panels a, b) and in CO_2_-saturated N_2_ (panel c). In panel a, relatively rapid changes during the first seconds of illumination are presented. In Fig. 3b, the slow induction kinetics in air over a course of 3 min are shown. A relatively low intensity of 630 nm actinic light (AL) was applied, which is sufficient for inducing almost 80% Fd reduction and a typical ‘Kautsky effect’ in Chl fluorescence. At this moderate light intensity, no donor side limitation of PS I is induced, so that P700 remains almost fully reduced during induction and maximal PC oxidation amounts to 25% only. Once after the first seconds of illumination the P-level of fluorescence and a peak of Fd reduction are reached, the Fd redox state and fluorescence yield appear to be almost perfectly correlated (Fig. 3b). Hence, under the given conditions, not only PS I, but also PS II is limited by the PS I acceptor side. All induction responses are considerably modified in the absence of molecular oxygen (Fig. 3c).

After dark-adaptation some key enzymes of the Calvin–Benson cycle downstream of Fd as well as the reversible ATP-ase are inactivated, so that following onset of illumination the pools of directly accessible acceptors are limited. The filling up of these pools is reflected by the rapid Fd reduction kinetics. Comparison of the Fd and fluorescence signals in Fig. 3a reveals that most of the Fd reduction occurs during the *early* plateau at the I-level of fluorescence yield. The I–P rise, which reflects the filling up of the PQ pool, sets in when about 70% of Fd is already reduced. Under the given conditions, fluorescence yield is mostly controlled by photochemical quenching, as higher quantum flux densities are required for development of nonphotochemical quenching.

The interpretation of the slow induction transients in Fig. 3b is more difficult, as the reactions downstream of Fd are extremely complex, involving numerous alternative pathways and feedback controls. Therefore, at this stage it would be premature to try interpreting all features of the slow induction kinetics. Even 75 years after the discovery of the ‘Kautsky effect’ (Kautsky and Hirsch 1931), the interpretation of the slow fluorescence induction transients has remained controversial (Stirbet and Govindjee 2016). However, one may be confident that with the new information on the Fd redox state and the concurrent information on P700 and PC, it may be possible to clarify some of the controversial aspects. While Chl fluorescence is a most powerful indicator of energy conversion in PS II (Papageorgiou and Govindjee 2004), its in vivo responses can be ambiguous due to various quenching mechanisms. It is a distinct advantage of the Fd signal that it is clearly defined by the Fd redox state and that it *directly* responds to the state of the PS I acceptor side.

The slow Fd redox changes in Fig. 3b suggest that after its initial reduction Fd is reoxidized in at least two waves, with the first wave corresponding to the so-called P–S fluorescence transient. The Fd reoxidation transients carry information on light activation of the reactions downstream of Fd, which may be assumed to respond to light activation of the reversible ATP-ase as well. The first wave of Fd reoxidation disappears upon removal of O_2_ together with the P–S fluorescence transient (see also Fig. 5 in Schreiber and Klughammer 2016), confirming a role of O_2_ reduction in an early step of light activation. Oxygen-dependent electron flow has been known for some time to play a crucial role during the induction of photosynthesis (Franck et al. 1969; Schreiber and Vidaver 1974; Allen 1975; Egneus et al. 1975; Radmer and Kok 1976; Marsho et al. 1979; Schreiber and Neubauer 1990; Asada 1999; Miyake 2010).

### Two phases in Fd reduction and evidence for a rapid cyclic pathway

**Fig. 4:**
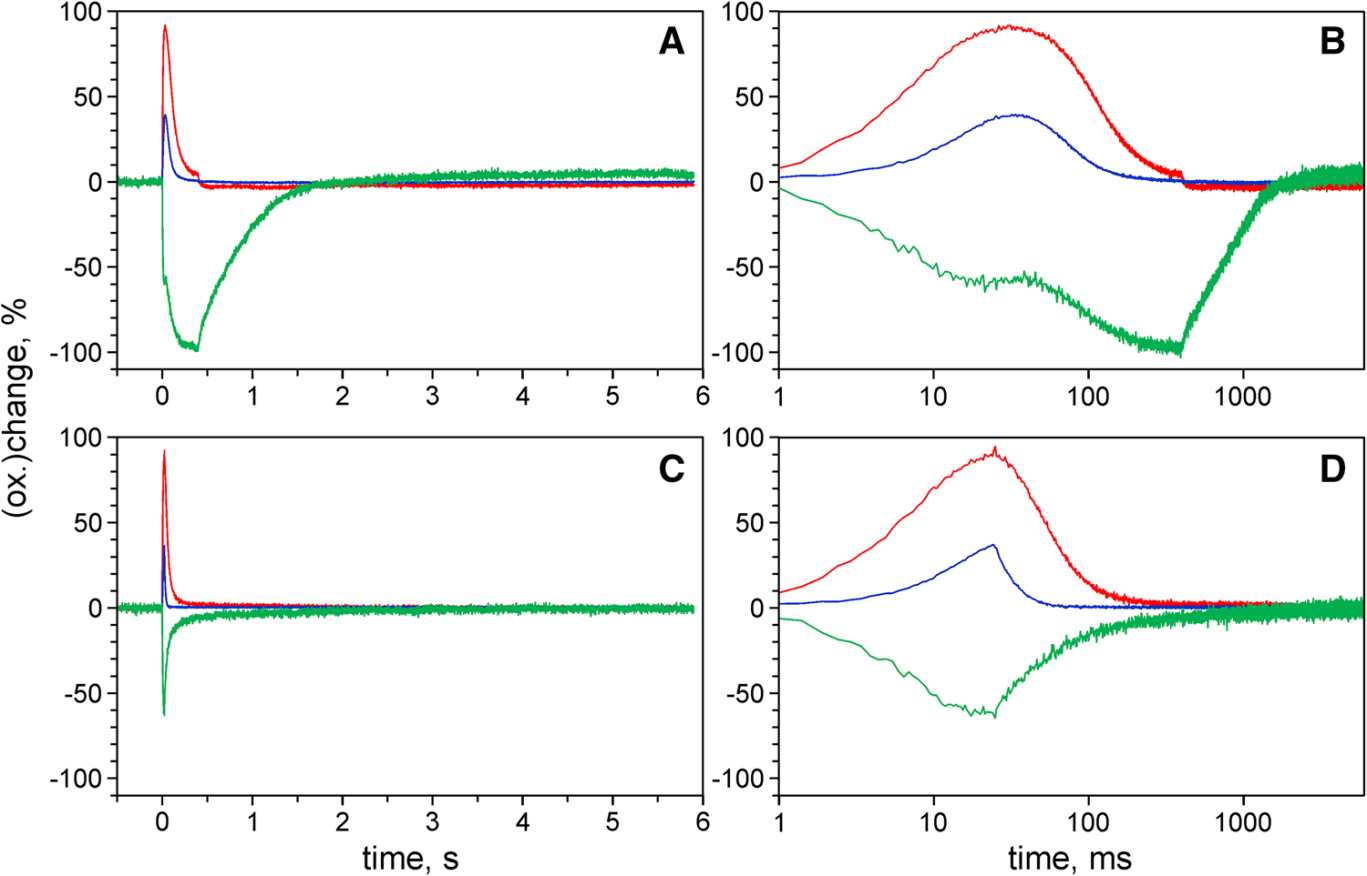
Redox changes of Fd (*green*), P700 (*blue*), and PC (*red*) induced by pulses of strong 630 nm light (1400 µmol m^−2^ s^−1^). **a**,** b** 400-ms pulse width. **c**,** d** 25-ms pulse width. Display with linear (**a**,** c**) and logarithmic (**b**,** d**) time scales. Dark-adapted intact ivy leaf. Averages of four recordings each with 2-min dark intervals between consecutive recordings

In the early part of the Fd reduction kinetics upon AL-on in Fig. 3a, two phases may be distinguished, which can be much better separated when the intensity of the actinic light that drives Fd reduction is increased. For the measurements of Fig. 4, strong light pulses (1400 µmol m^−2^ s^−1^ at 630 nm) with 400 ms (panels a, b) and 25 ms (panels c, d) widths were applied.

As clearly revealed by the logarithmic plot in Fig. 4b, upon onset of strong illumination Fd is reduced in two well-separated steps. The kinetics of the first step, which leads to about 60% Fd reduction, coincide with initial *oxidation* of PC (ca. 90%) and P700 (ca. 40%). Following an intermediary plateau, the second step of Fd reduction starts about 40 ms after AL-on, corresponding to the time during which the intersystem electron transport chain is filled up with electrons originating from water splitting in PS II. Consequently, Fd reduction during the second step coincides with the *rereduction* of P700 and PC.

It is worth pointing out that the first step of Fd reduction parallels *oxidation* of P700 and PC, whereas in the second step Fd reduction coincides with *reduction* of P700 and PC. Furthermore, the plateau phase of Fd reduction coincides with the peaks in oxidation of PC and P700 which do *not* get fully oxidized. These characteristics are in line with the following interpretation: Cessation of Fd reduction and PC oxidation at the end of the first step is caused by rapid cycling of electrons from Fd (red.) back to the donor side of PS I (CET). As CET depends on oxidized carriers in the intersystem electron transport chain, it becomes gradually suppressed when the chain becomes reduced by PS II (Allen 2003; Miyake 2010). With CET becoming suppressed, Fd reoxidation via CET ceases, so that Fd reaches full reduction in a second step.

This interpretation of the two-step Fd reduction is supported by comparison of the relaxation kinetics after a 400-ms pulse (panels a, b) and a 25-ms pulse (panels c, d). Termination of the 25-ms pulse coincides with the end of the plateau, shortly *before* the intersystem electron transport chain is filled up by electrons set free in PS II and rereduction of P700 plus PC sets in. Obviously, after the 25-ms pulse Fd reoxidation is much faster than after 400 ms, and Fd reoxidation is paralleled by the rereduction of P700 plus PC. Hence, under the given experimental conditions, the rate of Fd reoxidation may be considered a measure of the rate of CET. The rapid CET observed after 25-ms illumination of the dark-adapted sample at high PAR obviously is a *transient phenomenon*, which is disappeared after 400-ms illumination. Similar conclusions on the transient character of rapid CET were drawn by Joliot and Joliot (2005). The essential point in the context of the present communication is the demonstration of the *existence* of such rapid pathway of CET in intact leaves. It remains to be investigated to what extent this pathway is used by plants during steady-state illumination under natural environmental conditions.

### Determination of the Fd oxidation rate

In principle, the rate of Fd oxidation in any given light state can be measured with the Dual/KLAS-NIR spectrophotometer simply by monitoring the Fd redox state after AL-off. Fd is the first stable electron acceptor of PS I and in any stationary state of illumination its rate of reduction by PS I equals the overall rate of its reoxidation via various pathways. When AL is switched off, the initial slope of Fd oxidation is a measure of the overall oxidation rate. Depending on conditions, more or less recombination, LET (reduction of ferredoxin–NADP reductase, FNR, and of various alternative acceptors) as well as CET may contribute to this rate. An important peculiarity of Fd reoxidation by CET is that this is paralleled by P700 plus PC reduction (see, e.g., Fig. 4).

**Fig. 5:**
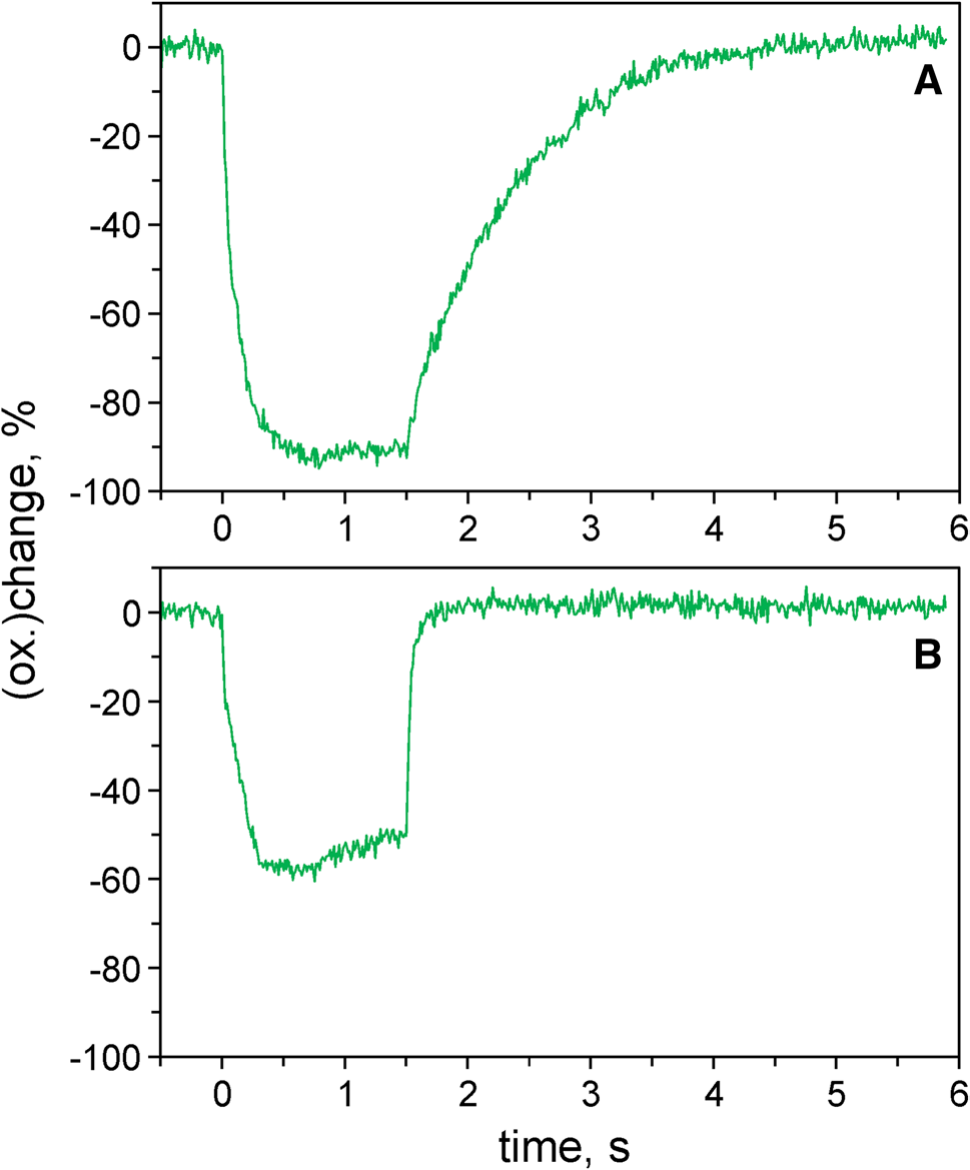
Reoxidation kinetics of Fd following a 1.5-s pulse of actinic illumination (300 µmol m^−2^ s^−1^) measured with an intact ivy leaf. **a** After 3 h dark-adaptation. **b** 1 min after termination of 4-min illumination at 40 µmol m^−2^ s^−1^. Single measurements

In practice, *direct* measurements of dark-relaxation kinetics upon switching-off of continuous AL are feasible only under conditions of weak or moderate light activation, because otherwise most of Fd will be oxidized and the oxidation rate of the remaining small fraction of reduced Fd will be rather high. An alternative approach, which is practicable also under conditions of Fd being mostly oxidized (e.g., under conditions of continuous illumination), makes use of a short pulse of light that causes transient Fd reduction without any significant change in the state of light activation. This approach can be applied for assessment of the Fd reoxidation rate after dark-adaptation and also after different extents of light activation. Examples of Fd reoxidation kinetics following short intense pulses of actinic light using a dark-adapted leaf were already presented in Fig. 4. In Fig. 5, the Fd reoxidation kinetics of an ivy leaf before (panel a) and after light activation (panel b) are compared. Light activation was induced by 4-min illumination at 40 µmol m^−2^ s^−1^ of 630 nm light.

A 1.5-s pulse of 300 µmol m^−2^ s^−1^ of 630 nm light was applied for inducing transient Fd reduction which was close to 100% in a dark-adapted sample. Such pulses could be applied every 5 min without changing the dark-inactive state. The Dual/KLAS-NIR software provides a routine for fitting the reoxidation kinetics with up to three exponentials, for which the amplitudes and time constants are determined. In the given example, satisfactory fits were obtained with just one exponential, yielding a time constant of 820 ms in the case of the dark-adapted leaf and 44 ms for the preilluminated leaf. Obviously not much light is required to lower the time constant of Fd oxidation substantially.

**Fig. 6:**
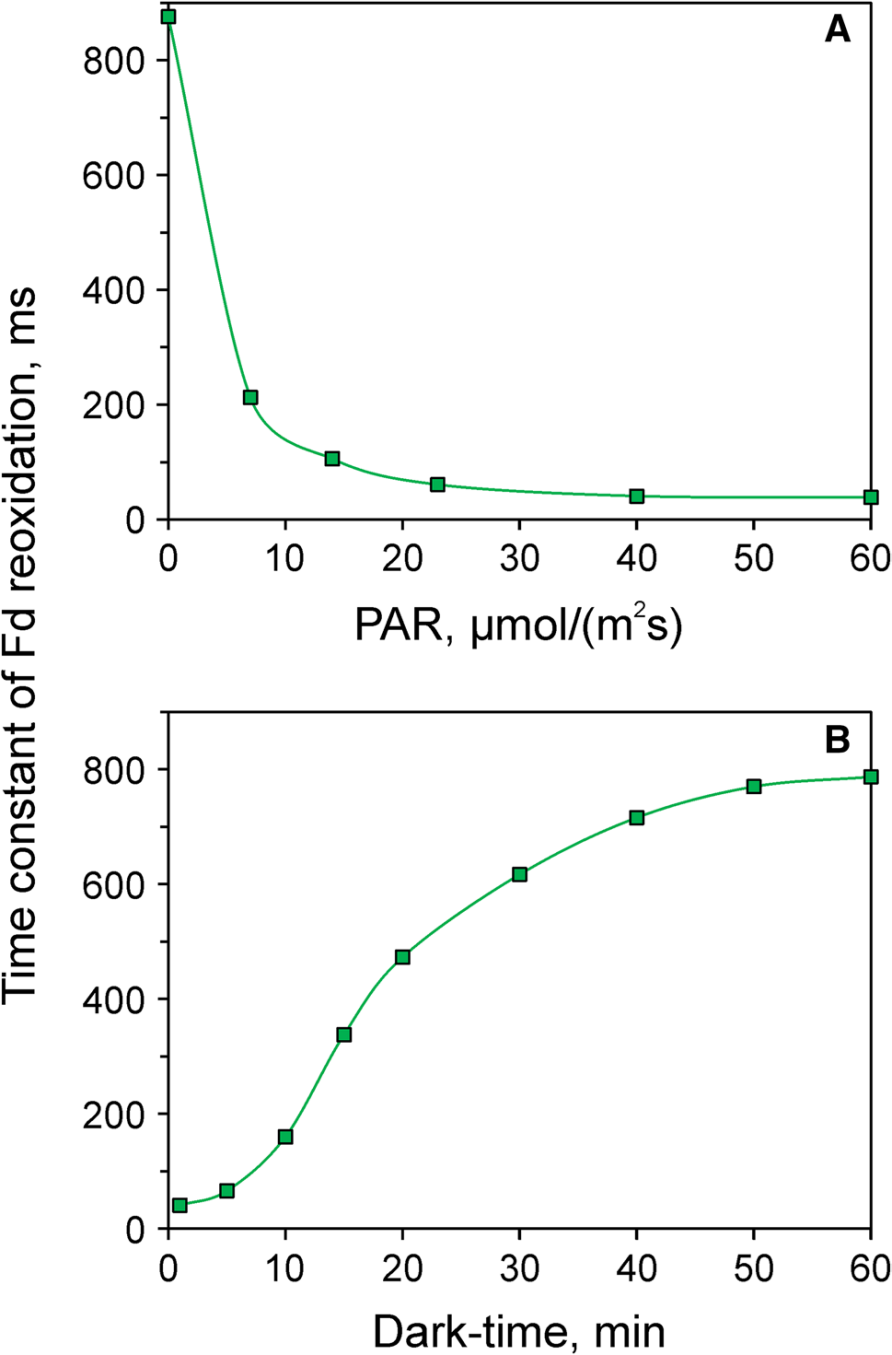
Time constant of Fd reoxidation as a function of the state of light activation. **a** Time constant plotted versus PAR applied during 4-min preillumination. **b** Time constant versus the dark-time after 4-min illumination at 60 µmol m^−2^ s^−1^. Application of a 1.5-s actinic light pulse (300 µmol m^−2^ s^−1^) for transient Fd reduction and consequent measurement of Fd reoxidation kinetics. Intact ivy leaf. Data from single recordings, as shown in Fig. 5

Measurements analogous to those of Fig. 5 were made after the leaf was preilluminated for 4-min periods at increasing light intensities, with 1 min of darkness between termination of preillumination and recording of the reoxidation kinetics. In Fig. 6a, the resulting time constants are plotted versus quantum flux density of PAR.

When the same leaf was darkened again, the observed dark-inactivation of Fd oxidation was rather slow, as shown in Fig. 6b, where the time constant of Fd oxidation is plotted versus the dark-time following 4-min illumination at 60 µmol m^−2^ s^−1^. Several phases can be distinguished in the dark-inactivation kinetics. During the course of an initial sigmoidal phase, which under the given conditions extended over ca. 10 min, the time constant was increased to about 200 ms. This phase was followed by a rise to about 500 ms within the next 10 min. The further rise to a saturated dark level of ca. 800 ms took about 40 min. The relative contributions of these phases were found to vary considerably between different species, depending on the physiological as well as ontogenetical state of the plant. Considering the complexity of the possible reactions downstream of Fd, at this stage it would be premature to attempt interpreting the various phases. In future work, not only light activation of the FNR and of the Calvin–Benson cycle should be considered, but also of various alternative pathways, like O_2_-dependent electron flow and CET as well as of the reversible ATP-ase.

### Siebke et al. (1991) revisited

An early attempt to obtain information on light activation of the enzymatic dark reactions at the PS I acceptor side was made by Siebke et al. (1991) who employed a standard PAM-100 with single beam 828 nm measuring light for monitoring P700 redox changes (Schreiber et al. 1988). Siebke et al. (1991) observed that upon onset of FR illumination an initial rapid phase of P700 oxidation was followed by a reductive phase (dip), before a second oxidative phase set in. Analysis of these transients led these authors to conclude that the applied FR illumination contained sufficient PS II light to rereduce P700 after the initial step of oxidation when the primary acceptor pool of PS I became exhausted. The secondary phase of P700 oxidation was explained by the opening of an ‘electron gate’ situated between PS I and the electron acceptor phosphoglycerate. Reduction of NADP during the initial phase of P700 oxidation showed that the electron gate was *not* identical to FNR, thus confirming previous conclusions of Neubauer and Schreiber (1989) based on Chl fluorescence measurements. While Siebke et al. (1991) did not consider that CET may affect the FR-induced P700 oxidation kinetics, later work by Joliot et al. (2004), Joliot and Joliot (2005), Joliot and Joliot (2006), and Talts et al. (2007) has provided strong evidence for involvement of CET, although some details of the FR-induced P700 transients have remained unexplained.

In the following experiments, we have revisited the P700 measurements of Siebke et al. (1991) taking advantage of the new technical means offered by the Dual/KLAS-NIR spectrophotometer. Not only do the deconvoluted Fd and PC signals provide additional, new information, but also the deconvoluted P700 signal gives more reliable information, as it is not ‘contaminated’ by PC and Fd changes.

**Fig. 7:**
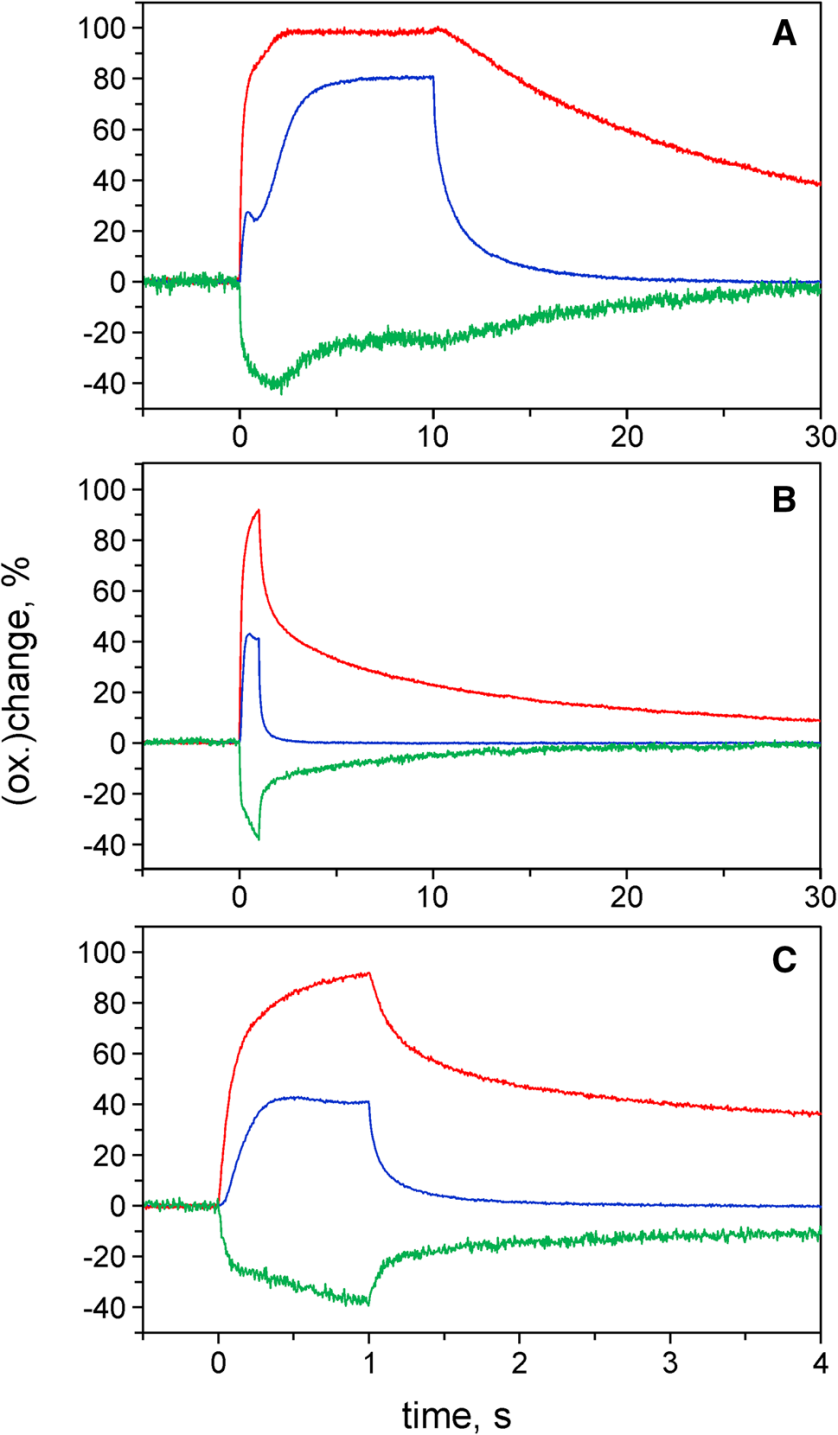
Redox changes of Fd (*green*), P700 (*blue*) and PC (*red*) upon FR-on and FR-off, measured with intact ivy leaf. FR, 400 µmol m^−2^ s^−1^ of 740 nm light. **a** 10-s FR illumination (single run). **b** 1-s FR illumination (average of five runs triggered with 2-min clock intervals), full time span of 30 s. **c** Horizontally zoomed part of **b** displayed with 4-s span

In Fig. 7, the deconvoluted redox changes of Fd, P700, and PC that are induced by strong 740 nm light in an intact ivy leaf are presented, corresponding to the measurements in Fig. 3 of Siebke et al. (1991). From the data in panel a, it is apparent that the second step of FR-induced P700 oxidation coincides with *reoxidation* of about half of the Fd reduced in the first step of P700 oxidation. Hence, it appears that the Fd (red.) which has accumulated during the first step of P700 oxidation (because initially no acceptors for its reoxidation were available) becomes oxidized along with P700 oxidation when the postulated opening of an ‘electron gate’ is induced about 2 s after onset of FR illumination. At the same time, however, this gate seems to be opened for part of PS I only. Upon FR-off after 10-s illumination, when stationary levels of about 80% P700 (ox.), close to 100% PC (ox.) and 25% Fd (red.) were reached, P700 rereduction was much faster than both PC rereduction and Fd reoxidation (panel a). Obviously, after 10-s FR there was a severe lack of electrons, as it took about 14 s for PC to become rereduced by 50%. At the same time also the reoxidation of the 25% Fd (red.) was very slow (t_1/2_ ca. 8 s).

On one hand, these data suggest that under the given conditions there was no link between the 25% fraction of Fd (red.) and the PC pool (i.e., no cyclic pathway, CET). On the other hand, for this fraction of Fd obviously also no effective linear electron transport pathway, LET, was open. This means that the apparent light activation, which supposedly occurred during the second step of P700 oxidation, was not reflected in the post-FR Fd reoxidation kinetics.

The relaxation kinetics changed dramatically when the FR was switched off already 1 s after FR-on, i.e., at the dip level following the first step of P700 oxidation (panels b, c). The P700 reduction was substantially speeded up (by about a factor of five) and rapid phases appeared in the relaxation kinetics of both PC reduction and Fd oxidation, with half-times in the order of 80 and 200 ms, respectively. Hence, the dip in P700 oxidation appears to be due to a backflow of electrons from Fd to PC and P700, suggesting that CET is involved. Notably, this type of CET gradually disappears at FR illumination times >1 s during the course of the second step of P700 oxidation, as the fraction of Fd involved in this reaction becomes oxidized (Fd oxidation starting about 2 s after FR-on).

Two different causes may be envisaged for parallel oxidation of P700 and Fd during the course of the second phase of P700 oxidation, namely

1. activation of Fd oxidation via LET, and
2. leakage of the electrons from CET to various alternative pathways, including O_2_-dependent electron flow, so that the reducing power for CET is gradually lost.

Whereas the slow Fd reoxidation kinetics upon FR-off are difficult to be reconciled with cause 1, the slow PC rereduction kinetics upon FR-off argue for cause 2. Therefore, it appears that cause 2 is mainly responsible for parallel oxidation of P700 and Fd, i.e., Fd reduction as well as P700 rereduction via CET are gradually suppressed due to an increasing lack of electrons. Hence, whereas the measurements of Fig. 4 have suggested suppression of CET-dependent Fd oxidation due to ‘overreduction’ caused by strong PS II light, the data in Fig. 7 argue for a suppression of CET due to ‘bleeding out of electrons.’ This interpretation is in line with the notion of proper ‘redox poising’ being required for CET (Heber 2002, Allen 2003; Miyake 2010).

The data in Fig. 7a suggest that electron leakage applies to about half of FR-reduced Fd only, whereas the other half appears to be well protected against rapid loss of electrons, arguing for the coexistence of *two fractions of Fd*, the redox states of which do *not* equilibrate. Furthermore, the data in Fig. 7b, c argue for two fractions of PS I, one of which shows rapid CET, while the other shows very slow relaxation kinetics of Fd and PC.

**Fig. 8:**
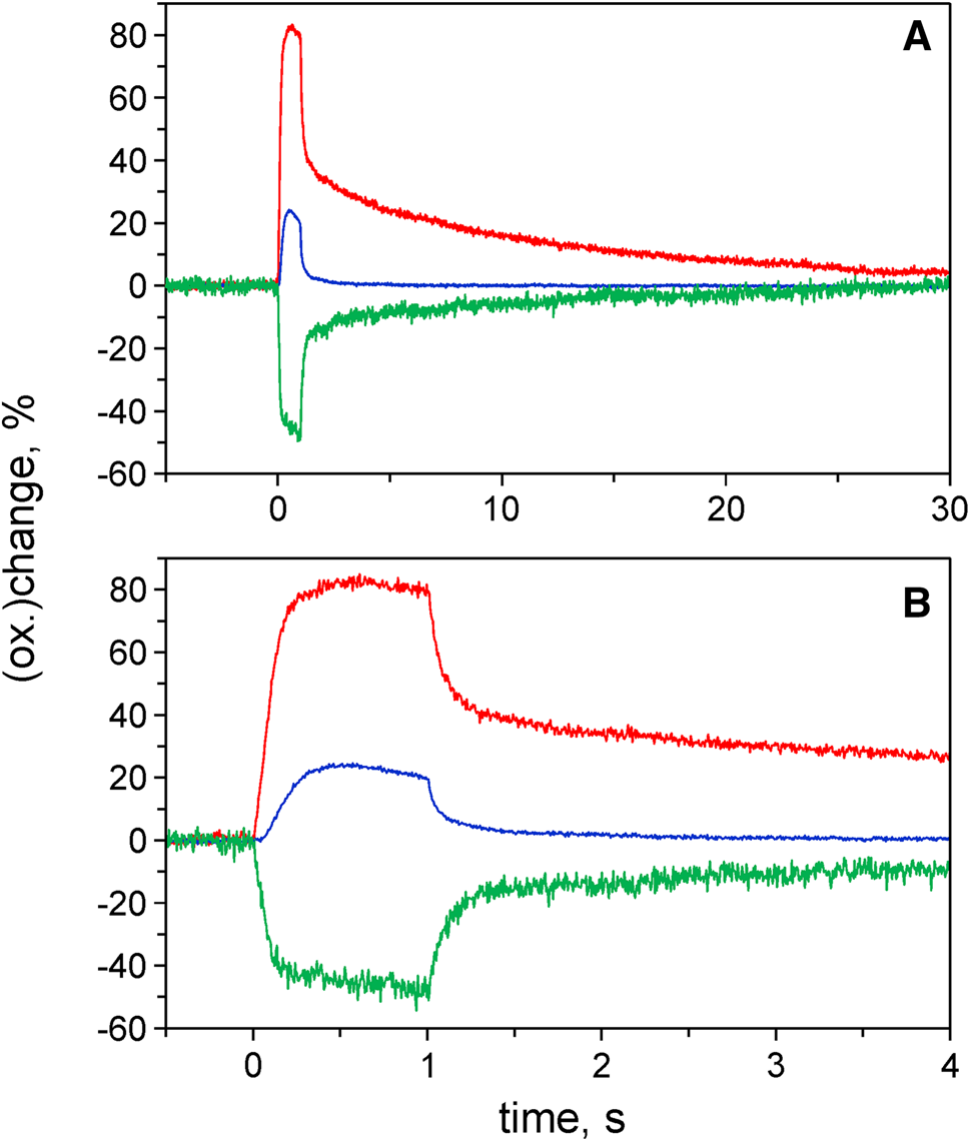
Redox changes of Fd (*green*), P700 (*blue*), and PC (*red*) upon 1-s FR illumination, measured with intact maize leaf (average of two runs triggered with 2-min clock interval). The same recording is presented with full 30-s time span (**a**) and horizontally zoomed with 4-s span (**b**). FR, 290 µmol m^−2^ s^−1^ of 740 nm light

Based on the rereduction kinetics of P700 and PC in Fig. 7c, the apparent equilibrium constants (K_app_) between P700 and PC can be estimated for the reactions reflected by the rapid and slow phases. For this purpose, the instrument software provides a ‘redox plot’ routine, with which P700(red.)/P700(ox.) can be plotted versus PC (red.)/PC (ox.) (see Supplementary Fig. 2 in Schreiber and Klughammer 2016). The slope of this plot corresponds to K_app_. Values of K_app_ = 22 and 114 are estimated for the rapid and slow phases of reoxidation, respectively. Similar data were obtained with other C3 leaves (not shown in the figures) and also with a C4 leaf (maize, see data in Fig. 8).

### Fd, P700, and PC responses in maize

With the evolution of C4 photosynthesis and of separate PS I (CET) in the BSC, optimal conditions for effective CET were created. In C4 leaves, redox equilibration between PS I (CET) and PS I (LET) is prevented by compartmentation, and the reducing power derived from water splitting in PS II is transferred from MC to BSC via the malate–oxaloacetate shuttle, which is light regulated (Rebeille and Hatch 1986; Scheibe 1990). On the other hand, in the case of C3 thylakoids, the notion of functional separation of PS I (CET) and PSI (LET) in segregated membrane domains (Anderson 1982; Albertsson 1995, 2001) to date has not been generally accepted. While Joliot and Joliot (2002), Kirchhoff et al. (2004), Joliot et al. (2004), as well as Joliot and Joliot (2005) have interpreted their results on the basis of the Albertsson domain model, Joliot and Joliot (2006) as well as Talts et al. (2007) did not find any evidence for the presence of two populations of PS I in C3 leaves that possibly could reflect functional differences of PS I in stroma and grana thylakoids.

The data in Fig. 7 have provided new evidence for heterogeneous pools of Fd and PC in C3 leaves that are *not* in redox equilibrium with each other. For further evaluation of this evidence, in Fig. 8 a measurement with maize analogous to that with ivy in Fig. 7b, c is presented.

The 1-s FR responses in maize (Fig. 8a) are similar to those in ivy (Fig. 7b). The amplitude of the rapid phase of Fd reoxidation upon FR-off, however, is larger in maize, which agrees with the notion that in this C4 leaf a larger fraction of PS I is engaged in CET. The biphasic reoxidation kinetics of Fd are remarkably antiparallel to the rereduction kinetics of PC, showing that the electrons for rereduction of the PC oxidized by FR originate from Fd reduced by FR. Hence, these data support the interpretation of the data in Fig. 7b, c as reflecting CET in the C3 leaf. The coexistence of two types of Fd with largely differing reoxidation rates upon FR-off argues for separation of these two types of Fd by compartmentation, not only in the C4, but also in the C3 leaf. The same is true for the PC serving as electron donor of both types of PS I.

Based on the rereduction kinetics of P700 and PC in Fig. 8b, the apparent equilibrium constants between P700 and PC can be estimated for the reactions reflected by the rapid and slow phases, similarly as described above for the analogous experiment with ivy. In the case of maize, values of K_app_ = 8 and 126 are estimated for the rapid and slow phases of reoxidation, respectively. The maize data suggest that the low K_app_ applies for PS I (BSC) and the high K_app_ for PS I (MC). As was shown by Drepper et al. (1996) and discussed by Kirchhoff et al. (2004), the midpoint redox potential of PC bound to PS I is shifted to a more positive value compared to that of free PC. Consequently, the equilibrium constant between P700 and PC decreases from a value of about 85 (for free PC) to about 10 (for bound PC). Apparently in PS I (BSC), the redox equilibration between PC and P700 is determined by PC bound to the reaction center complex, which means that the concentration of free PC does not play any significant role.

Whereas the role of CET in C3 photosynthesis is still under debate, there has been general consensus that CET plays an important role in C4 photosynthesis, where two types of PS I operate in mesophyll cells (MC) and bundle sheath cells (BSC), with PS I (BSC) running a separate type of CET and a small fraction of PS II (BSC) presumably serving a redox poising function (Albertsson 2001). Crucial roles of NDH-dependent CET in C4 photosynthesis were demonstrated by analyzing mutant maize (Peterson et al. 2016) and Flaveria plants (Ishikawa et al. 2016). Kimata and Hase (1989) and Hanke and Hase (2008) have reported on specific expression of Fd isoproteins in MC and BSC. While Fd I was found in both MC and BSC, Fd II is expressed in BSC only. The total amount of Fd in BSC was about two times higher than in MC. In view of these physiological peculiarities, the responses of Fd, P700, and PC in C4 leaves may be expected to show more pronounced CET-linked characteristics than in C3 leaves. Hence, it should be possible to obtain information on these characteristics by comparing the phenomenology of light-induced responses of C4 and C3 leaves.

**Fig. 9:**
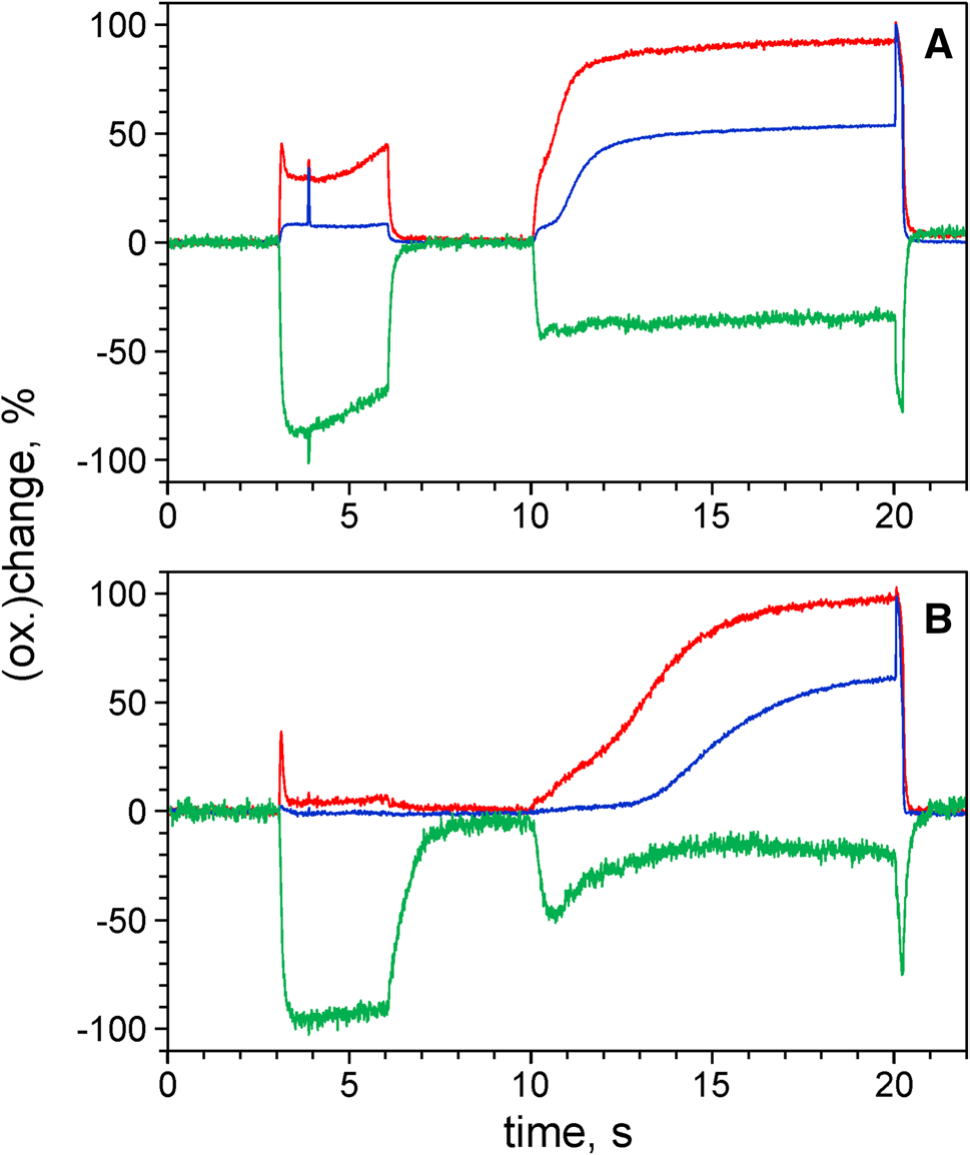
Comparison of routine measurements for determination of 100% responses of Fd (*green*), P700 (*blue*), and PC (*red*) with leaves of maize (**a**) and sunflower (**b**). Illumination with 300 µmol m^−2^ s^−1^ of 630 nm light (3–6 s) followed by illumination with 290 µmol m^−2^ s^−1^ of 740 nm light (10–20 s). 30-ms MT pulses with 10,000 µmol m^−2^ s^−1^ of 630 nm light applied at 4 and 20 s. Single script runs

In Fig. 9, the light-induced responses of dark-adapted maize and sunflower leaves are compared, using a standard illumination program for induction of maximal redox changes of Fd, P700, and PC. These are script-controlled measurements that are carried out routinely with every new leaf sample in order to define maximal signal changes associated with 100% redox changes of Fd, P700, and PC (Klughammer and Schreiber 2016, see also “Materials and methods” section). Maximal Fd reduction is assessed during an initial 3-s illumination with 300 µmol m^−2^ s^−1^ of 630 nm light and maximal oxidation of P700 and PC is determined at the end of a 10-s FR illumination period. Saturating multiple turnover pulses (MT) of 630 nm light are applied for inducing maximal Fd reduction at 4 s and maximal oxidation of P700 and PC at 20 s.

While the overall phenomenology of the observed Fd, P700, and PC responses is similar in maize and sunflower, some differences are apparent that are likely to relate to differences in C4 and C3 photosynthesis:

1. In maize, Fd reoxidation during the initial 3-s AL period is more pronounced, being accompanied by substantial PC oxidation, whereas PC in sunflower stays almost fully reduced after an initial spike of oxidation.
2. The 30-ms MT applied at 4-s results in distinct spikes of Fd reduction as well as of P700 and PC oxidation in maize, but not in sunflower.
3. The reoxidation kinetics of Fd upon AL-off at 6 s are much faster in the case of maize compared with sunflower.
4. The FR-induced oxidation of P700 and PC is much more rapid in maize than in sunflower.
5. While in maize the level of FR-induced Fd (red.) remains almost constant during the 10-s illumination period, in sunflower a pronounced decline is observed.
6. The quasi-stationary final level of P700 oxidation is somewhat lower in maize than in sunflower.

The observed differences suggest that in maize the reactions downstream of Fd (and possibly also the reversible ATP-ase) do not inactivate in the dark to the same extent as in sunflower (points 1–4 above). Furthermore, the apparent light activation, which in sunflower leads to reoxidation of Fd *during* FR illumination (see Fig. 7), does not seem to play much of a role in maize (points 5–6 above).

The FR intensity of 290 µmol m^−2^ s^−1^ applied in the measurements of Fig. 9 is relatively high, allowing a rate of charge separation of approximately 20 electrons s^−1^, as can be estimated from the initial slopes of PC oxidation and Fd reduction. The turnover rate of PS II induced by the same FR may be considered negligibly small, as deduced from the rate of the FR-induced fluorescence increase in the presence of DCMU (not shown in the figures). Therefore, when in the measurements of Fig. 9 large fractions of P700 remain quasi-stationarily reduced after 10 s of strong FR illumination, this means that in a fraction of PS I either charge separation is prevented by lack of oxidized Fd or P700 becomes rapidly rereduced by CET. The fact that 100% P700 oxidation can be readily induced by a saturating MT strongly argues in favor of CET. Consequently, the fraction of P700 that remains reduced during strong stationary FR illumination (equivalent to the amplitude of the P700 oxidation spike induced by a saturating MT) appears to be closely related to the fraction of PS I being capable of relatively rapid CET.

### Test for estimation of CET

The FR-induced responses displayed in Fig. 9 were measured under conditions that favor CET against LET, as the leaves were dark-adapted and sufficient electrons were pumped into the intersystem chain by the initial pulse of actinic light that was applied for assessment of 100% Fd reduction. With the help of light activation, conditions can be created that are more representative for steady-state photosynthesis under natural conditions. As was shown in Fig. 6, not much light is required for inducing high rates of Fd reoxidation by LET. Any CET that can be detected even after light activation may be considered to be relevant for photosynthesis under normal physiological conditions.

**Fig. 10:**
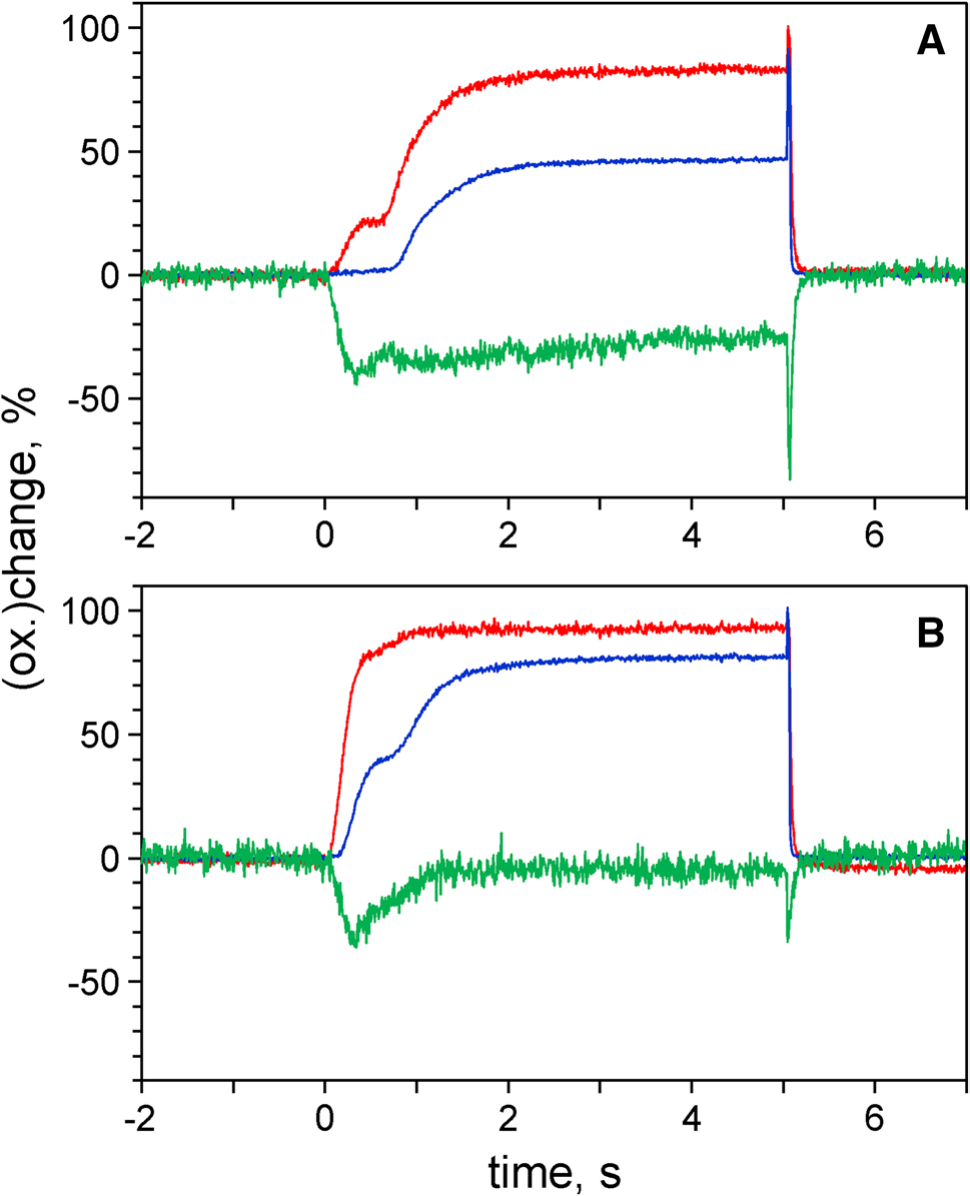
Estimation of the fraction of PS I operating in the cyclic mode in light-activated leaves with the help of a 30-ms saturating MT after 5-s illumination with strong FR light (290 µmol m^−2^ s^−1^). **a** Maize; estimated CET fraction 50%. **b** Sunflower; estimated CET fraction 20%. Single runs

In Fig. 10, the FR-induced responses of Fd, P700, and PC in moderately *preilluminated* maize and sunflower leaves are compared. The leaves were preilluminated for 10 min at 14 µmol m^−2^ s^−1^ 630 nm AL. A 5-s pulse of FR was applied 13 s after termination of preillumination. At the end of the FR pulse a 30-ms MT was applied for assessment of 100% P700 oxidation. In these light-activated samples, stationary values of FR-induced oxidation of P700 and PC were already reached after 3 s. While in maize stationary P700 oxidation was similar to that under the dark-adapted conditions of Fig. 9, in sunflower light activation of LET had led to a substantial increase of P700 oxidation. The values of stationary P700 reduction were 50% for maize and 20% for sunflower.

**Fig. 11:**
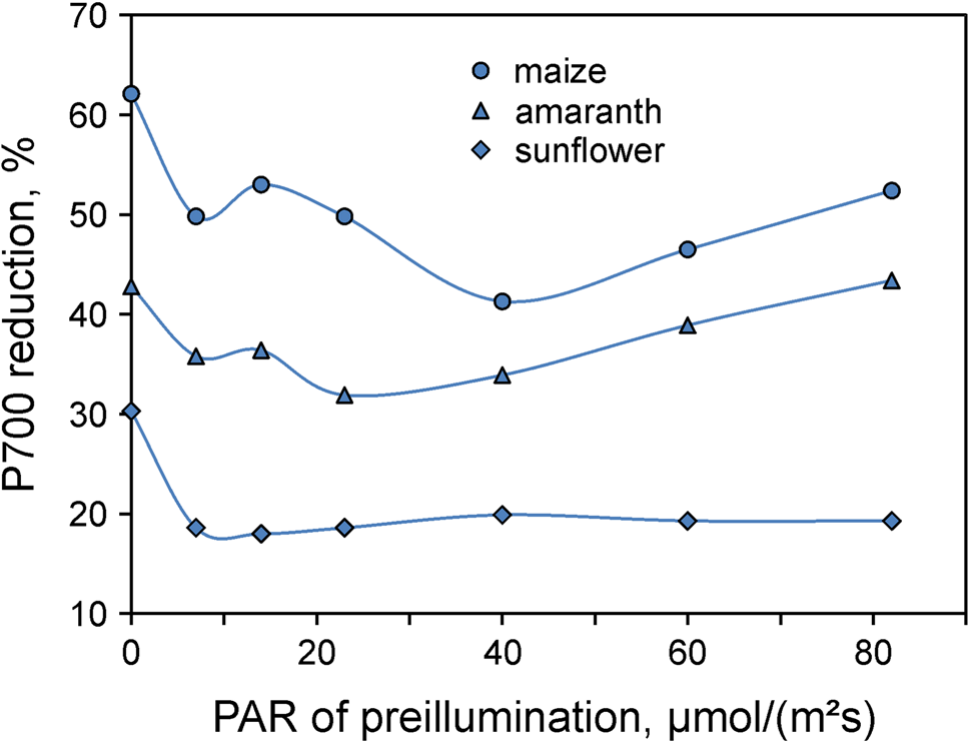
Extent of P700 reduction after 5-s illumination with strong FR light in maize, amaranth, and sunflower as a function of the intensity of 10-min preillumination by 630 nm light. The P700 (red.) amplitudes are considered a proxy of the fraction of PS I engaged in CET. Data obtained from single recordings as shown in Fig. 10

The thus determined quasi-stationary values of P700 reduction after 5-s illumination by strong FR light show some variation depending on leaf species and physiological parameters. As already apparent by comparison of the responses in Figs. 9 and 10, one of these parameters is the state of light activation. In Fig. 11, the percentage of P700(red.) after 5-s illumination by strong FR light (i.e., the postulated estimate of CET) is plotted as a function of the intensity of 10-min preillumination by 630 nm light for maize, amaranth (*A. retroflexus*), and sunflower. In all three species, the estimated apparent CET declines upon light activation using the lowest PAR setting (7 µmol m^−2^ s^−1^). While in sunflower after the initial drop the apparent CET assumes a relatively stable value around 20%, in maize and amaranth (a C4 plant) the apparent CET shows variations at considerably higher levels, which appear likely to reflect regulatory processes of C4 photosynthesis.

The data in Fig. 11 suggest that the fraction of PS I engaged in CET, which shall be denoted PS I (CET), is a dynamic parameter showing considerable variations during dark–light induction. The rationale of the applied measuring routine relies on three assumptions:

1. The fraction of reduced P700 in the presence of strong FR mostly reflects PS I (CET), where P700 is effectively rereduced.
2. Due to the preceding light activation, P700 in PS I (LET) is mostly oxidized in presence of strong FR.
3. There is no redox equilibration between PS I (CET) and PS I (LET).

These assumptions probably are not strictly fulfilled, so that the CET test in its present form should be considered to give rough estimates for PS I (CET) only. For example, PS I (CET) is likely to be somewhat underestimated, because part of P700 (CET) could be oxidized in spite of effective CET. This depends on the location of the rate limiting step in CET, the pool size of the electron carriers between Fd and P700, and also on the effective equilibrium constant between PC and P700. On the other hand, if some P700 in PS I (LET) were reduced, in spite of light activation, this would result in some overestimation of PS I (CET). Future work must show, how large the resulting errors are, to what extent they compensate each other and whether corrections are possible.

## Concluding discussion and outlook

It was the aim of the present communication to present evidence supporting the good news that Fd redox changes can be reliably measured in intact leaves. For this purpose, a dedicated measuring system was developed that is capable of deconvoluting the relatively small transmittance changes of Fd in the NIR from considerably larger transmittance changes of P700 and PC (Klughammer and Schreiber 2016). Deconvolution relies on small differences in the oxidized minus reduced absorbance spectra of Fd, P700, and PC in the 780–1000 nm wavelength range (Figs. 1, 2). By suitable choice of 4 wavelength pairs in this range, four difference signals with maximal information on the three redox components are obtained. The key for deconvolution of the mixed difference signals consists in determination of the changes induced by *selective* redox changes of either Fd, P700, or PC (Klughammer and Schreiber 2016). The ‘differential model plots’ (or ‘model spectra’) obtained with a dark-green ivy leaf proved valid for a variety of other higher plant leaves (Schreiber and Klughammer 2016), so that deconvolution has become a standard routine carried out in the background by the computer. Consequently, redox changes of Fd, P700, and PC can be recorded continuously in parallel with Chl fluorescence.

The presented data have demonstrated Fd redox changes under a variety of conditions, using various types of intact leaves. For the interpretation of Fd redox changes, the simultaneously measured redox changes of P700 and PC as well as Chl fluorescence changes proved important. In this context, it is worth pointing out that the P700 signals measured with the Dual/KLAS-NIR spectrophotometer differ from all previously measured ‘P700’ signals, as for the first time they are free of overlapping changes of PC and Fd, which depending on conditions may contribute substantially to 820 nm transmittance changes (see Fig. 2). This aspect is emphasized by the data in Fig. 3, where the induction of large changes of PC and Fd is not accompanied by any change of the P700 redox state.

In the past, several attempts were made to deconvolute P700 and PC redox changes from NIR transmittance changes. Oja et al. (2003) and Talts et al. (2007) applied a mathematical model of the PS I donor side based on the assumption of equilibrium between the P700/P700^+^ and PC/PC^+^ redox couples. Kirchhoff et al. (2004) determined the differential absorption coefficients required for deconvolution of P700 and PC from PS I- and PC-enriched preparations, coming to the conclusion that equilibration between PC and P700 is *not* homogenous throughout thylakoid membranes. Joliot and Joliot (2006) deconvoluted P700 and PC from dual-wavelength transmittance measurements, assuming fixed contributions of the two components to the ΔI/I measured at each of these wavelengths. However, more recently Joliot and Johnson (2011) returned to single wavelength 820 nm measurements, considering that absorption changes at 820 nm give an acceptable approximation of the P700 redox state. Whether this is true or not strongly depends on the conditions, as may be concluded from the deconvoluted data presented in the present communication. In any case, the deconvoluted PC signal carries important information of its own, which differs strongly from that of deconvoluted P700 and can be of considerable help with the interpretation of Fd changes.

The presented data were focused on dark–light induction and light–dark relaxation kinetics, as under these conditions most pronounced Fd redox changes can be observed. The dark–light induction kinetics of Fd reduction presented in Fig. 3 are an almost perfect mirror image of the simultaneously measured induction of Chl fluorescence. Antiparallel changes essentially persisted after removal of molecular oxygen, which eliminated a rapid phase of Fd reoxidation in parallel with the elimination of the P–S transient in fluorescence. These data provide strong evidence that electron transfer to O_2_ is an early event during induction, before light activation of various enzymatic reactions of the Calvin–Benson cycle.

As Fd, P700, and PC could be deconvoluted with high time resolution (1 ms/data point in standard recordings), it was possible to follow the rapid redox changes upon onset and termination of strong illumination. The outcome of these measurements demonstrates the value of simultaneously measured Fd, P700, and PC kinetics, particularly when a possible involvement of CET is discussed. The return of an electron from reduced Fd at the PS I acceptor side to the PS I donor side should be indicated by the concerted oxidation of Fd and reduction of P700 plus PC. Due to the higher oxidation potential of P700, P700 reduction generally is faster than PC reduction.

In Fig. 4, data were presented that support an involvement of CET during the first 100 ms after onset of strong illumination driving both photosystems. This conclusion is primarily based on the observation of antiparallel kinetics of Fd oxidation and PC reduction, when strong illumination already was terminated 25 ms after light-on. This type of CET appears to set in, when a substantial amount of total Fd has become reduced, while PC is mostly oxidized and seems to be suppressed again when the PQ pool becomes reduced by PS II. In this way, the observed two steps of light-induced Fd reduction can be explained.

As was shown in Figs. 5 and 6, the Fd oxidation kinetics can be reliably evaluated and information on light activation can be obtained. It has been known for long that some enzymes of the Calvin–Benson cycle (Ziegler and Ziegler 1965; Buchanan 1980; Scheibe 1990) as well as the reversible ATP-ase (Bakker-Grunwald and van Dam 1974; Schreiber 1980) are inactivated in the dark and become reactivated upon illumination. Reduction of thioredoxin and ensuing transfer of SH groups are known to be involved. However, not much details are known on the in vivo mechanisms. The notion of ferredoxin–NADP reductase (FNR) being subject to such reductive light activation (Carillo et al. 1981) appears difficult to be reconciled with the observation of single-turnover flash-induced NADP reduction in dark-adapted intact chloroplasts (Schreiber and Klughammer 2009; Kauny and Sétif 2014). Following dark-adaptation, Fd reoxidation after a short Fd-reducing light pulse is slow (half-times in the order of 0.5–1 s). Very few light (ca. 10 µmol m^−2^ s^−1^) is required to enhance the rate by an order of magnitude. At present, it is not clear which of the many potential pathways of Fd oxidation become activated at what light intensity. In view of the data in Fig. 3 (elimination of first step of Fd reoxidation by removal of O_2_), in future work the role of molecular oxygen in an early step of light activation should be investigated. However, it is known that also activation of the reversible ATP-ase requires very few light (Schreiber and Del Valle-Tascon 1982; Kramer and Crofts 1989). The data in Fig. 4 suggest that Fd reoxidation by CET does not require activation, but rather depends on proper ‘redox poising’ and the presence of oxidized PC in particular. When the light by which reduction of Fd was induced is switched off in a state of largely reduced PC, Fd reoxidation by CET is minimized and dominated by more or less activated LET.

From the experiment of Fig. 7, it can be concluded that *transiently* favorable conditions for CET are generated, when Fd reduction is induced by FR light in a dark-adapted leaf. While the P700 response in this measurement was very similar to that reported by Siebke et al. (1991), a somewhat different interpretation is suggested by the simultaneously measured Fd and PC responses. The explanation offered by Siebke et al. (1991) of cessation of P700 oxidation upon exhaustion of a primary acceptor pool, followed by a second step of P700 oxidation when the ‘electron gate’ to a secondary pool is opened, is questioned by the fact that the rate of Fd reoxidation at the end of the 10-s FR illumination period is very low. Obviously, the FR illumination has not opened any gate for the Fd (red.) that remains after 10-s FR. Instead, rapid Fd reoxidation was observed after 1-s FR (i.e., at the time where P700 had reached the intermediary level), being paralleled by rapid phases of P700 and PC reduction, thus strongly arguing in favor of CET being involved. CET is induced when Fd (red.) coincides with a high concentration of PC^+^. It declines again with the exhaustion of the PS I donor pool and with the unavoidable electron leakage to alternative acceptors, including O_2_.

An aspect that has been controversially discussed during more than 20 years and still has not been clarified is the question of whether the PS I located in the stroma lamellae functionally differs from that located in the grana margins and end membranes, as originally proposed by Anderson (1982) and Albertsson (1995). The low PS II/PS I ratio in the stroma lamellae, accompanied by an unusually high content of NADP dehydrogenase, suggests that PS I in this surrounding is mainly engaged in CET. On the other hand, PS I located in the grana margins and end membranes is close to PS II, so that its engagement in LET appears most likely. Joliot et al. (2004), after having discovered a highly efficient cyclic pathway during the first seconds of illumination, argued that their finding ‘obligatorily requires that the carriers involved in the cyclic and linear chains be structurally separated in order to limit the rate of electron exchange between these two pathways.’ However, Joliot and Joliot (2006) came to the contrary conclusion that CET and LET compete for the reoxidation of Fd that freely diffuses in the stromal compartment. Future work must show whether this conclusion can be maintained when analogous measurements are carried out with deconvolution of Fd, P700, and PC.

The relaxation kinetics upon FR-off presented in Fig. 7b, c reveal a fraction of PS I characterized by rapid Fd reoxidation paralleled by a rapid phase of PC rereduction and another fraction of PS I displaying much slower Fd reoxidation and PC rereduction. Hence, at least under the given conditions of a FR-illuminated dark-adapted leaf, two fractions of PS I can be distinguished. Such distinction becomes difficult, if not impossible, when LET becomes light activated and the Fd reoxidation kinetics are correspondingly speeded up.

An undisputable case of PS I heterogeneity is given in C4 photosynthesis, where PS I (MC) primarily serves LET and PS I (BSC) is engaged in CET. The maize data presented in Figs. 8, 9, 10, and 11 on one hand show many similarities to the corresponding ivy and sunflower data. On the other hand, the particular responses that in the C3 leaves were considered to reflect CET (Fig. 7) were distinctly more pronounced in maize (Fig. 8). Comparison of the data in Figs. 9 and 10 suggests that considerable CET occurs upon strong FR illumination in dark-adapted sunflower, which is partially suppressed by light activation. The question arises whether the estimated 20% PS I (CET) that remain in sunflower after light activation may be related to the about 20% stroma lamellae determined by Albertsson (2001) in a large variety of C3 thylakoids. In this context, it appears worth mentioning that also in Fig. 1 of Joliot and Joliot (2006) an approximately 20% fraction of P700 is not oxidized by strong FR light. While the significance of this fraction was not discussed, the response following preillumination by a saturating 200-ms light pulse was taken as evidence for *all* PS I centers contributing to CET. This finding, however, does not rule out the existence of a fraction of PS I (CET) located in the stroma lamellae of C3 with properties analogous to those of PS I (BSC) in C4. Future work will have to show whether the simple CET-test demonstrated in Fig. 10 can generally serve to estimate the size of such fraction of PS I (CET), which may be considered a type of ‘constitutive’ PS I (CET) that is not suppressed by light activation. Hanke and Hase (2008) have characterized two types of Fd in leaves of *Arabidopsis*. It is tempting to speculate that the minor Fd isoprotein could be related to PS I (CET).

The fraction of PS I (CET) indicated by the CET test depends on the intensity of preillumination, as was shown in Fig. 11. In both C3 and C4 leaves, the fraction of P700 that remained reduced during strong FR illumination declined with preillumination at low 630 nm intensity, in agreement with the expected light activation of LET. While in sunflower at higher intensities this fraction remained close to 20%, it showed considerable variations in maize and amaranth depending on the intensity of preillumination. In view of the fact that PS I (CET) in C4 is dominated by PS I (BSC), the observed variations appear likely to be caused by variations in electron donation from stroma reductants to the PQ pool (Asada et al. 1992, 1993), presumably via the NDH.

It is important to note that the conditions under which evidence for a rapid pathway of CET was obtained (in Figs. 4, 7, 8) were far from the conditions under which photosynthetic electron transport normally takes place. The same is true for analogous experiments of other researchers (Joliot et al. 2004; Talts et al. 2007; Laisk et al. 2010). Therefore, while such experiments demonstrate the *existence* of such pathways, they do not say much about their relevance in vivo. As has been previously pointed out by Heber and Walker (1992), Heber (2002), Allen (2003), and Miyake (2010), CET requires proper redox poising, which means that there must be an excess of electrons at the PS I acceptor side coexisting with a substantial fraction of oxidized carriers at the PS I donor side. While it may be assumed that these prerequisites are assured in the case of PS I (BSC) in C4 even after light activation, it remains to be clarified to what extent this is true for PS I in C3 and whether it may apply for a PS I (CET) fraction in the stroma lamellae only. Practically all arguments that have been put forward for an important physiological role of CET in C3 (Heber and Walker 1992, Heber 2002, Breyton et al. 2006; Joliot and Johnson 2011) apply for O_2_-dependent electron flow as well (Schreiber and Neubauer 1990; Hormann et al. 1994; Schreiber et al. 1995; Park et al. 1996; Asada 1999; Hideg et al. 2008; Miyake 2010). Possibly O_2_-dependent electron flow and CET are linked, with the former providing proper redox poising of the latter (Miyake 2010).

The potential reactions at the acceptor side of PS I under in vivo conditions are very complex. Until very recently, most information on these reactions were obtained by *indirect* measuring techniques, relying on light-induced oxidation and rereduction of P700 or Chl fluorescence changes. With the new possibility of measuring redox changes of Fd, P700, and PC simultaneously, the way is opened for a more direct and profound analysis. Nevertheless, in view of the complexity of the involved reactions, it should be expected that some time will pass until a clear view of the regulation of the reactions at ‘the end of the line’ has evolved. In this respect, the present situation is similar to that in 1986, when PAM fluorometry was introduced, which for the first time allowed to measure Chl fluorescence in natural daylight (Schreiber 1986; Schreiber et al. 1986; Schreiber and Bilger 1987). In spite of hundreds of PAM-based studies, the complexity of in vivo Chl fluorescence changes still is not fully understood (see, e.g., Stirbet and Govindjee 2012). Based on experience from the analysis of complex fluorescence changes, it may be predicted that computer-assisted modeling will play an important role in the interpretation of the complex responses of Fd, P700, and PC. It is a distinct advantage of Fd, P700, and PC redox changes that these can be interpreted in a straight forward way, without the need of differentiating between photochemical and various forms of nonphotochemical quenching.

Various models of CET have been proposed in the past. While many genetic and biochemical studies have indicated that CET is mediated by NDH- and PGR5-dependent pathways (see review by Shikanai 2007), to date no details on the involvement of Fd in vivo is available. In some models, it is assumed that electrons return from Fd (red.) to the PS I donor side via the low-potential chain of the cyt b_6_f complex (Joliot and Joliot 2006; Laisk et al. 2010; Joliot and Johnson 2011). In this case, parallel measurements of cyt f and cyt b_6_ redox changes in intact leaves may be expected to complement the information obtained with simultaneous Fd, P700, and PC measurements. Such measurements are in preparation using a Kinetic LED Array Spectrophotometer (KLAS-100) in the 510–570 nm range, capable of deconvoluting in vivo cytochrome redox changes (Klughammer et al. 1990).

## Abbreviations

AL: Actinic light
BSC: Bundle sheath cell
CET: Cyclic electron transport in PS I
Chl: Chlorophyll a
COB: Chip-on-board array of light emitting diodes
DCMU: 3-(3,4-Dichloro-phenyl)-1,1-dimethylurea
Fd: Ferredoxin
F_X_, F_A,_ F_B_: FeS clusters at acceptor side of PS I core complex
FluoML: Pulse-modulated 540 nm fluorescence measuring light
FNR: Ferredoxin–NADP reductase
FR: Far-red light
ΔI/I: Change of transmittance
KLAS: Kinetic LED array spectrophotometer
LED: Light emitting diode
LET: Linear electron transport
MC: Mesophyll cells
MAP: Mehler-ascorbate-peroxidase cycle
MT: Multiple turnover light pulse driving both photosystems
NDH: NADPH dehydrogenase
NIR: Near-infrared light
NIRmax: Maximal NIR transmittance changes of Fd, P700, and PC
PAM: Pulse amplitude modulation
PAR: Quantum flux density of photosynthetically active radiation
PC: Plastocyanin
PQ: Plastoquinone
P–S: Chl fluorescence decline from peak to first stationary level
P700: Reaction center chlorophyll of photosystem I
